# Clarifying the Inflammatory Roots of Perianal Abscess: A Commentary on Mendelian Randomization Insights From 91 Circulating Proteins

**DOI:** 10.1002/hsr2.71193

**Published:** 2025-08-18

**Authors:** Jonathan James O. Canete

**Affiliations:** ^1^ Department of Innovation and Sustainability, De La Salle University Manila Philippines

Dear Editor,

This article is in recognition of the recently published research article, “Unveiling the Intricate Causal Nexus Between 91 Circulating Inflammatory Proteins and Perianal Abscess Through a Comprehensive Bidirectional Two‐Sample Mendelian Randomization Analysis” by Wang et al. This study presents a significant and timely investigation into the causal relationship between systemic inflammation and the development of perianal abscess—a condition that remains a clinical challenge due to its complex pathogenesis and high recurrence rates.

The authors' application of a bidirectional two‐sample Mendelian randomization (MR) analysis to explore this relationship offers critical insights into the inflammatory pathways underlying perianal abscess. By utilizing summary‐level data from genome‐wide association studies (GWAS) for 91 circulating inflammatory proteins and perianal abscess risk, the study transcends the limitations of observational research that often struggle with confounding and reverse causation [1]. This methodological rigor not only strengthens the causal inference but also highlights the potential of genetic epidemiology to clarify disease mechanisms.

A key strength of the study is its identification of specific inflammatory proteins—namely interleukin‐6 (IL‐6), tumor necrosis factor‐alpha (TNF‐α), and C‐reactive protein (CRP)—as causally associated with an increased risk of perianal abscess. These findings underscore the critical role of systemic inflammation in the pathophysiology of the disease and suggest potential therapeutic avenues that target these inflammatory mediators. Considering the substantial morbidity associated with perianal abscess, these insights are invaluable for both preventive strategies and treatment optimization.

Moreover, the authors' implementation of bidirectional MR analyses is commendable, as it rigorously assesses the possibility of reverse causation. Their finding that genetic liability to perianal abscess does not significantly influence circulating levels of inflammatory proteins further validates the unidirectional causal relationship—supporting the hypothesis that systemic inflammation precedes and potentially drives disease onset rather than being a mere consequence of the abscess itself. This distinction has important clinical implications for developing targeted interventions aimed at modulating inflammatory processes to mitigate disease risk.

While the study's design is robust, it also raises important considerations for future research. The potential for horizontal pleiotropy, where genetic variants influence outcomes through pathways unrelated to the exposure of interest, remains a challenge inherent to MR studies. Although the authors utilized sensitivity analyses—including MR‐Egger regression and weighted median approaches—to address this concern, residual confounding cannot be completely excluded. Further efforts to refine genetic instruments and incorporate multi‐omics approaches, such as transcriptomics and proteomics, could enhance the specificity and validity of these causal estimates [2].

Another dimension worth exploring is the interaction between these inflammatory proteins and other established risk factors for perianal abscess, such as microbiome composition, local immune responses, and environmental influences. Integrating longitudinal cohort studies with multi‐layered biological data would offer a more nuanced understanding of disease progression and the potential for early detection and intervention.

The study's findings also call attention to the need for a paradigm shift in the management of perianal abscess. Current clinical approaches often prioritize surgical drainage and antibiotic therapy, with limited focus on the underlying inflammatory processes. The identification of specific inflammatory proteins as causal factors supports the integration of anti‐inflammatory treatments—such as cytokine inhibitors—into standard care protocols. Such an approach could potentially reduce disease incidence, improve healing outcomes, and decrease recurrence rates.

In addition, while the study's reliance on GWAS data from European ancestry populations provides valuable insights, it also highlights the importance of validating these findings in diverse populations to ensure broader generalizability and equitable clinical translation. Addressing this gap in future research could enhance the inclusivity and applicability of these findings across global healthcare settings.

In general, this study makes a substantial contribution to our understanding of perianal abscess pathogenesis by employing a rigorous bidirectional MR approach to identify systemic inflammation—mediated by specific circulating proteins—as a causal factor. The findings lay a strong foundation for future research into targeted anti‐inflammatory interventions and emphasize the need for integrated, patient‐centered care models that address the underlying biological mechanisms of disease. I commend the authors for their valuable work and look forward to further discussions on translating these insights into clinical practice to improve outcomes for patients with perianal abscess.

## Author Contributions

**Jonathan James O. Canete:** conceptualization, writing – review and editing, resources.

## Acknowledgments

This manuscript utilized QuillBot to support drafting, refining, and formatting while ensuring alignment with academic standards. All content was critically evaluated and revised by the authors to maintain originality, accuracy, and integrity.

## Conflicts of Interest

The author declares no conflicts of interest.

## Transparency Statement

The [lead author/manuscript guarantor] affirms that this manuscript is an honest, accurate, and transparent account of the study being reported; that no important aspects of the study have been omitted; and that any discrepancies from the study as planned (and, if relevant, registered) have been explained.

## Data Availability

The data that support the findings of this study are openly available in Wiley Online at https://doi.org/10.1002/hsr2.70803.
